# An Analysis of the Gut Microbiota and Related Metabolites following PCSK9 Inhibition in Statin-Treated Patients with Elevated Levels of Lipoprotein(a)

**DOI:** 10.3390/microorganisms12010170

**Published:** 2024-01-15

**Authors:** Jose A. Caparrós-Martín, Patrice Maher, Natalie C. Ward, Montserrat Saladié, Patricia Agudelo-Romero, Stephen M. Stick, Dick C. Chan, Gerald F. Watts, Fergal O’Gara

**Affiliations:** 1 Wal-yan Respiratory Research Centre, Telethon Kids Institute, Perth, WA 6009, Australia; 2 Curtin Health Innovation Research Institute (CHIRI), Curtin University, Perth, WA 6102, Australia; 3 Dobney Hypertension Centre, Medical School, The University of Western Australia, Perth, WA 6009, Australia; 4 The University of Western Australia, Perth, WA 6009, Australia; 5 Department of Respiratory Medicine, Princess Margaret Hospital for Children, Perth, WA 6008, Australia; 6 Medical School, Faculty of Health and Medical Sciences, The University of Western Australia, Perth, WA 6009, Australia; 7 Cardiometabolic Service, Departments of Cardiology and Internal Medicine, Royal Perth Hospital, Perth, WA 6000, Australia; 8 BIOMERIT Research Centre, School of Microbiology, University College Cork, T12 XF62 Cork, Ireland

**Keywords:** PCSK9, alirocumab, statins, LDL, cholesterol, gut microbiota, bile acids, short chain fatty acids, atherosclerotic cardiovascular disease

## Abstract

Background. Atherosclerotic cardiovascular disease (ASCVD) is a leading cause of global mortality, often associated with high blood levels of LDL cholesterol (LDL-c). Medications like statins and PCSK9 inhibitors, are used to manage LDL-c levels and reduce ASCVD risk. Recent findings connect the gut microbiota and its metabolites to ASCVD development. We showed that statins modulate the gut microbiota including the production of microbial metabolites involved in the regulation of cholesterol metabolism such as short chain fatty acids (SCFAs) and bile acids (BAs). Whether this pleiotropic effect of statins is associated with their antimicrobial properties or it is secondary to the modulation of cholesterol metabolism in the host is unknown. In this observational study, we evaluated whether alirocumab, a PCSK9 inhibitor administered subcutaneously, alters the stool-associated microbiota and the profiles of SCFAs and BAs. Methods. We used stool and plasma collected from patients enrolled in a single-sequence study using alirocumab. Microbial DNA was extracted from stool, and the bacterial component of the gut microbiota profiled following an amplicon sequencing strategy targeting the V3-V4 region of the 16S rRNA gene. Bile acids and SCFAs were profiled and quantified in stool and plasma using mass spectrometry. Results. Treatment with alirocumab did not alter bacterial alpha (Shannon index, *p* = 0.74) or beta diversity (PERMANOVA, *p* = 0.89) in feces. Similarly, circulating levels of SCFAs (mean difference (95% confidence interval (CI)), 8.12 [−7.15–23.36] µM, *p* = 0.25) and BAs (mean difference (95% CI), 0.04 [−0.11–0.19] log10(nmol mg^−1^ feces), *p* = 0.56) were equivalent regardless of PCSK9 inhibition. Alirocumab therapy was associated with increased concentration of BAs in feces (mean difference (95% CI), 0.20 [0.05–0.34] log10(nmol mg^−1^ feces), *p* = 0.01). Conclusion. In statin-treated patients, the use of alirocumab to inhibit PCSK9 leads to elevated levels of fecal BAs without altering the bacterial population of the gut microbiota. The association of alirocumab with increased fecal BA concentration suggests an additional mechanism for the cholesterol-lowering effect of PCSK9 inhibition.

## 1. Introduction

Atherosclerotic cardiovascular disease (ASCVD) is the leading cause of death and morbidity worldwide. Elevated circulating levels of low-density lipoprotein cholesterol (LDL-c) is a major risk factor for ASCVD [1], and thus they are therapeutically targeted in primary and secondary prevention [2]. Statins with or without ezetimibe effectively lower LDL-c concentration and reduce the risk of ASCVD [3,4,5,6]. Lipoprotein(a) [Lp(a)] is an LDL-like particle that is a genetically determined independent risk factor for ASCVD [7]. Monoclonal antibodies targeting the protein convertase subtilisin-like/kexin type 9 (PCSK9) can effectively lower plasma LDL-c and to a lesser extent Lp(a) concentrations [8,9]. PCSK9 is a protein involved in the degradation of LDL receptors. PCSK9 inhibition using monoclonal antibodies results in enhanced clearance of LDL particles from the bloodstream by promoting LDL receptor recycling [10], and it is a recommended therapy for those high-risk individuals experiencing limited response to statin therapy [8].

Over the past few years, multiple studies examining the symbiotic microorganisms residing in the human intestinal tract have revealed a clear association between these bacterial communities and the development of ASCVD (reviewed in [11]). Notably, bacterial metabolites originating from the gut such as trimethylamine (TMA), which serves as a precursor to trimethylamine-n-oxide (TMAO), have been implicated in the development of ASCVD [12]. Thus, circulating levels of TMAO are associated with an elevated risk of cardiovascular events and mortality [13,14], while the utilization of specific inhibitors targeting TMA bacterial metabolism has shown a reduction in the risk of thrombus formation in animal models [15]. Accumulative evidence also indicates the involvement of additional gut bacteria-related metabolites, including short chain fatty acids (SCFAs) and bile acids (BAs), in the pathophysiology of ASCVD [16,17]. Short-chain fatty acids are metabolic by-products produced during the fermentation of dietary fiber by specific groups of microorganisms, particularly bacteria from genera such as *Clostridium*, *Eubacterium*, and *Bacteroides* [18]. These fatty acids have a significant impact on the host by influencing both the inflammatory response and metabolic status [18]. Bile acids, on the other hand, are cholesterol metabolites involved in lipid digestion. Beyond their role in digestion, BAs also serve as signaling molecules, playing important roles in immunity and metabolism [19,20], as well as in modulating the gut microbial communities [21,22]. Bile acids, initially synthesized in the liver as primary BAs, such as cholic acid and chenodeoxycholic acid, are conjugated with taurine or glycine, stored in the gall bladder, and released into the duodenum post meal [20]. Within the gastrointestinal tract, they undergo biotransformation by various members of the gut microbiota resulting in the formation of secondary BAs like deoxycholic acid and lithocholic acid [21,22]. A proportion of these secondary BAs is recycled by being reabsorbed in the distal intestine and transported through the portal vein into the liver where they are conjugated and stored [20]. Gut bacteria-induced alterations in the BAs pool influence the activation of BAs receptors like FXR or TGR5, which play roles in both immune and metabolic homeostasis [23], including the regulation of cholesterol and BA metabolism [24,25,26,27,28,29].

The involvement of gut microbiota and its associated metabolites in the progression of ASCVD highlights the importance of addressing not only the physiological triggers (e.g., cholesterol metabolism) but also the production of microbial bioactive metabolites influencing ASCVD pathology, as well as any proinflammatory bacteria that may exacerbate the condition. Drugs targeting cholesterol metabolism, such as statins or ezetimibe, have been shown to influence the intestinal microbiota as well as the pools of both BAs and SCFAs [30,31,32,33]. Thus, in a proof-of-concept study, we demonstrated that treatment with statins of otherwise healthy mice fed with normal diet, was associated with alterations in the composition and function of the gut microbiota, as well as with a transcriptional dysregulation of genes involved in lipid and glucose metabolism [30]. These pleiotropic effects of statins were tentatively explained by the statin-mediated activation of the nuclear receptor Pxr, which is also involved in the modulation of BA metabolism [30,34]. Additionally, a recent observational study found that statin therapy was associated with a lower prevalence of the Bact2 enterotype, a gut bacteria community type prevalent in individuals with obesity that is also associated with systemic inflammation [31]. Alterations in microbial diversity and function following treatment with statins could be explained by direct antibacterial effects of statins associated with the enteral administration of this medication [35]. However, these effects could also stem from the dysregulation of the metabolism of BAs associated with the alteration of cholesterol synthesis [30]. To investigate this further, we conducted an observational study involving individuals with poor response to statins, who received alirocumab, a subcutaneously administered monoclonal antibody targeting PCSK9. By using medication delivered via parenteral, we bypassed any potential impact on the gut microbiota. This approach allowed for us to evaluate whether modulation of cholesterol metabolism alters the ecology of the stool-associated bacterial biota, along with the levels of bacterial-associated metabolites BAs and SCFAs.

## 2. Materials and Methods

### 2.1. Cohort and Sample Collection

In this observational study, we opportunistically evaluated the fecal microbiota and its associated metabolites in patients enrolled in a single-sequence study aimed at assessing the effects of alirocumab, a PCSK9 neutralizing antibody, on the metabolism of apolipoprotein(a) [9]. Patients in this study had elevated lipoprotein(a) (Lp(a) > 0.5 g L^−1^) and were on long-term statin therapy [9]. Of the 21 subjects enrolled in the original study [9], 18 provided stool samples and agreed to participate in this ancillary study. Stool samples for fecal microbiome analysis were collected in polypropylene plastic containers (Sarstedt, Nümbrecht, Germany), frozen after collection, and transferred to Linear Clinical Research (Perth, Australia), where they were stored at −80 °C until processing. We collected the baseline stool samples during the kinetic study that was performed prior to administering the PCSK9 neutralizing antibody alirocumab [9]. On-treatment samples were collected during the kinetic study done at Week 12 while subjects were receiving alirocumab [9]. Venous blood was collected after a 14 h fast as described in the original study [9]. The study was approved by a national ethics committee (Bellberry Ltd., Eastwood, Australia), application number 2016-08-622-A-7, and all subjects provided informed consent.

### 2.2. Isolation of Microbial DNA from Stool Samples, 16S Metabarcoding, Sequencing of the Amplicon Pools, and Processing of the Sequencing Data

DNA was extracted from approximately 80 mg of homogenized stool using the QIAamp Fast DNA Stool kit (QIAGEN, Hilden, Germany) including a bead-beating step using 0.1 mm diameter zirconia/silica beads as we previously described [30,36]. We processed a negative extraction control alongside the patients’ specimens to identify reagent-related contaminants. The stool-associated bacterial component of the microbiota was profiled by sequencing a PCR amplicon encompassing the V3–V4 region of the 16S rRNA gene at Genewiz (Beijing, China) using V3 MiSeq chemistry (Illumina, San Diego, CA, USA) in a 2 × 300 bp run. In this project, we obtained a total count of 7,953,138 reads (sum of forward and reverse reads), distributed between 36 stool DNA extracts, and one negative extraction control. Raw data quality was assessed using FastQC and MultiQC [37]. Sequencing accuracy was good, with 89% of the reads having a mean Phred-like Q-score greater than or equal to 30. Sequencing files were quality-filtered and paired-end joined using Trimmomatic [38] as we described previously [39]. Quality-based processing and merging resulted in 3,173,814 paired-end reads (average length 449 bp), with a good base calling accuracy (92% of sequences with mean Phred-like Q-score greater than or equal to 35). Joint reads were clustered at 98% sequence identity using VSEARCH (Version 2.15.1) [40], and then assigned to taxonomy using the SILVAngs pipeline [41] and the last release of the SILVA SSU taxonomy (138.1) as we previously described [30,39]. Sequencing data were assigned to 664 Operational Taxonomic Units (OTUs). Computational processing of the taxonomic table involved the removal of singletons, reads classified as Chloroplast (mean relative abundance (standard deviation), 0.00006% (0.00009)), Mithocondria (0.00003% (0.0002)), and no classified reads (0.0007% (0.0004)). We also removed those OTUs representing less than 0.01% across all samples. While some reads in our dataset were initially classified into OTUs representing Archaea (Table S1), these specific OTUs were subsequently eliminated during this filtering process, as they represented less than 0.01% of the total abundance in the original dataset. Consequently, following the elimination of these low-abundance reads, only OTUs representing bacteria remained. We also removed one OTU representing 61% of the reads in the negative extraction control, which was assigned to the *Acinetobacter* taxon. Additional potential contaminants associated with the sample processing steps were identified and eliminated using the R package *decontam* [42]. This analysis detected 3 OTUs assigned to the *Staphylococcus*, *Bosea* and *Ochrobactrum* taxa, which in this dataset were likely representing environmental contaminants (Table S2). These findings are consistent with a previous study reporting contaminating sequences associated with environmental taxa such as *Acinetobacter*, *Ochrobactrum* or *Bosea* in negative extraction controls [43]. After performing these filtering steps, we obtained a taxonomic table representing the relative abundance for 170 OTUs. We used Procrustes analysis to confirm that the filtering steps described above did not significantly impact the overall structure of the unfiltered dataset (Table S3 and Figure S1).

### 2.3. Quantitative Profiling of Bile Acids and Short Chain Fatty Acids

We extracted bile acids (BAs) from stool samples following our previously published protocol [30,44]. Blood short chain fatty acids (SCFAs) were extracted from 100 µL of plasma and profiled as we previously described [30,36,44]. Because plasma sample stocks were exhausted for some patients, we assessed circulating levels of SCFAs in 19 samples. Among these, 16 samples were paired, representing both the baseline and on-treatment timepoints collected from 8 patients. Circulating levels of BAs were obtained from 100 µL of plasma using liquid–liquid extraction with 2 volumes of acetonitrile. Plasma BAs were analyzed for all the patients in this study, except for one patient for whom we did not have enough plasma samples from both baseline and on-treatment visits. Samples were vortexed and centrifuged at 13,500 rpm for 10 min at 4 °C. Then, 150 µL of supernatant were dried under nitrogen stream and reconstituted in 100 µL of 50% methanol in water. Bile acids were profiled using a mass MSMS instrument (Agilent QTOF 6540, San Diego, CA, USA) following our previously published method [44,45]. For identification of the different classes of BAs, we used the following pure standards to create a mass spectral–retention time library; taurocholic acid (Santa Cruz, sc220189, Dallas, TX, USA), taurolithocholic acid (Cayman Chemicals, 17275, Ann Arbor, MI, USA), taurochenodeoxycholic acid (Steraloids, C1162-000, Newport, RI, USA), tauroursodeoxycholic acid (Steraloids, C1052-000), glycolithocholic acid (Steraloids, C1435-000), glycochenodeoxycholic acid (Steraloids, C0962-000), glycoursodeoxycholic acid (Steraloids, C1025-000), glycocholic acid (Steraloids, C1927-000), glycodeoxycholic acid (Steraloids, C1087-000), ursodeoxycholic acid (Steraloids, C1020-000), cholic acid (Sigma-Aldrich, C1129, St. Louis, MO, USA), deoxycholic acid (Sigma-Aldrich, D2510), lithocholic acid (Sigma-Aldrich, L6250), chenodeoxycholic acid (Sigma-Aldrich, C1050000). For SCFA and BA quantification, blood and stool samples were spiked with internal standards before proceeding with the extraction protocol as we previously described [36,44,45].

### 2.4. Statistical Analyses

Statistical analysis was performed in R (Version 4.0.2) using built-in functions. For the linear model presented in this study, we confirmed the assumptions of normal distribution and homoscedasticity of residuals, and the absence of influential data points using regression diagnostics. We compared gut alpha diversity and the concentrations of both SCFAs and BAs between baseline and on-treatment samples using a paired *t*-test, following the confirmation of data distribution normality through the Shapiro–Wilk test. Principal component analysis model was built using the R package mixOmics [46]. PERMANOVA analysis and estimation of alpha diversity was performed using the functions of the R package vegan. To identify bacteria taxa differentially regulated between baseline and on-treatment samples, we used ANCOM-BC, which accounts for the compositionality of 16S amplicon sequencing data as well as for differences in sequencing depth, which is typically observed in amplicon sequencing datasets [47]. We used Dirichlet Multinomial Mixtures to identify community-based bacterial clusters or endotypes associated with response to alirocumab [48]. Following widespread scientific consensus, we set the cut-off for statistical significance at 0.05.

## 3. Results

### 3.1. Study Cohort

Briefly, we carried out a single-sequence study of the effect alirocumab on the ecology of the stool-associated bacterial biota and the profiles of SCFAs and BAs (baseline versus 12 weeks on treatment). All patients entered a 2-week run-in, diet-stabilizing period, at the end of which they were administered PCSK9 monoclonal antibody therapy for 12 weeks (alirocumab SC 150 mg every 2 weeks) [9]. Advice was given to patients to continue an isocaloric diet and maintain medication and physical activity constant until the end of the post-treatment period. All patients in this substudy were on maximally tolerated and stable statin therapy as prescribed in the clinic: rosuvastatin (40 mg day^−1^, n = 10; 10 mg day^−1^, n = 1; 5 mg day^−1^, n = 2), atorvastatin (20 mg day^−1^, n = 2), pravastatin (40 mg day^−1^, n = 1; 20 mg day^−1^, n = 1), and fluvastatin (80 mg day^−1^, n = 1); fourteen patients were also on a stable dose of ezetimibe (10 mg) [9]. All eighteen patients in this substudy were on aspirin (100 mg) and four on anti-hypertensive medication [9]. As indicated in the clinical study, patients were either diagnosed with subclinical atherosclerosis or had experienced at least one event related to atherosclerotic cardiovascular disease [9]. Patient demographics for this cohort are shown in Table 1. On average, the patients were middle age and non-obese. The effect of alirocumab on body mass index, blood pressures, plasma lipid and lipoprotein concentrations have been previously reported [9]. Briefly, body mass index, diastolic and systolic blood pressures did not alter significantly during the intervention (*p* > 0.05 for all) [9]. Alirocumab significantly lowered total cholesterol, triglyceride, LDL-cholesterol, apoB and Lp(a) concentration (*p* < 0.05 for all) [9]. Alirocumab was well tolerated in these patients with no adverse effect reported [9].

### 3.2. Alirocumab Is Not Associated with Alterations in the Taxonomic Profiles of the Fecal-Associated Bacterial Biota

We profiled the bacterial component of the fecal microbiota using an amplicon sequencing approach (Figure S2A). At phylum level, the taxonomic profiles were dominated by operational taxonomic units (OTU) belonging to the phyla Bacillota (formerly Firmicutes, mean relative abundance (standard deviation), 51.5% (18.6)), Bacteroidota (formerly Bacteroides, 40.5% (17.2)), and Pseudomonadota (formerly Proteobacteria, 5.4% (8.4)) (Figure S2B). At a genus level, an OTU assigned to the *Bacteroides* taxon was the more abundant feature in our cohort (30.3% (15.3)) (Figure S2C). In some patients, we noticed an increase in OTUs representing taxa from the phylum Pseudomonadota (*Enterobacter* and *Escherichia-Shigella*) in the on-treatment stool specimens (Figure S2A). However, in our cohort, the proportion of Pseudomonadota in feces was largely equivalent between baseline and on-treatment samples (paired *t*-test, *p* = 0.25).

**Figure 1:**
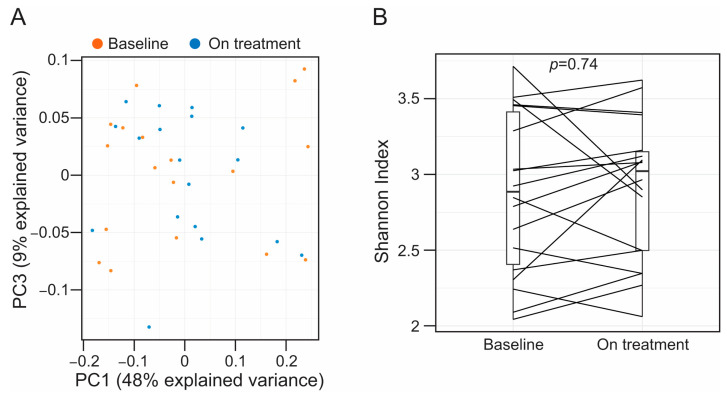
(**A**) Projection of the first and third principal components (PC) of the principal component analysis model. We chose to represent PC1 and PC3 as it provides a clear visualization of the results obtained from the PERMANOVA analyses. However, PC2 and PC3 explained a similar portion of the variance in our dataset, 13% and 9%, respectively, and we obtained a very similar plot when representing PC1 and PC2 (see Figure S3). Each dot represents individual fecal specimens, which are colored based on whether they were collected at baseline (orange) or on treatment (blue). (**B**) Boxplots showing alpha diversity metrics (Shannon index) in paired fecal samples. Samples collected from the same subject are connected with straight lines. For statistical inference, we used a paired t-test, and the observed *p*-value is shown in the graph.

Alirocumab treatment did not have an effect on overall gut microbiota composition (permutational multivariate analysis of variance (PERMANOVA): pseudo *F* = 0.41, R^2^ = 0.01, *p* = 0.89) (Figure 1A and Figure S3). Similarly, treatment with alirocumab was not associated with changes in alpha diversity of the stool bacterial communities (paired *t*-test, *p* = 0.74) (Figure 1B). Differential abundance analysis using ANCOM-BC did not identify OTUs differentially regulated after alirocumab therapy. Likewise, cluster analysis using Dirichlet Multinomial Mixtures did not identify different community-based clusters, suggesting the absence of different stool-associated bacteria endotypes in our patient cohort. On the contrary, based on the sum of squares (73% explained by subject), most of the community-level differences in our dataset were associated with individual variability (PERMANOVA: pseudo *F* = 2.87, R^2^ = 0.73, *p* = 0.0001) (Figure S4).

### 3.3. Alirocumab Is Linked to Changes in the Profiles of Gut Bacteria-Associated Metabolites

**Figure 2:**
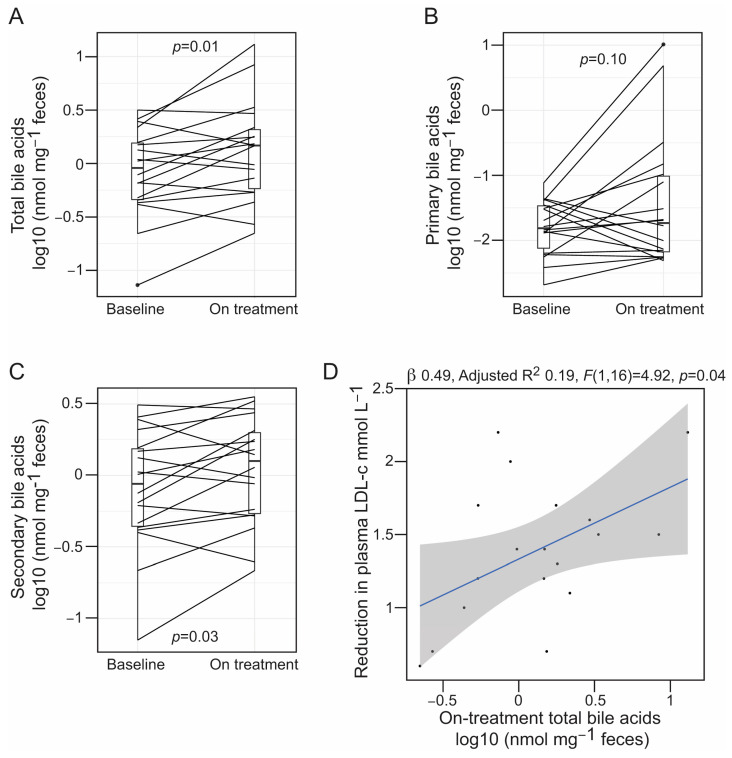
(**A**–**C**) Boxplots representing the concentration (log10 transformed) of total bile acids (**A**), and primary (**B**) and secondary (**C**) bile acids in fecal samples collected at baseline and on treatment. Samples from the same patient are connected using straight lines. The following primary bile acids were detected in feces: cholic acid, taurocholic acid, chenodeoxycholic acid, taurochenodeoxycholic acid and glycochenodeoxycholic acid. The following secondary bile acids were detected in feces: deoxycholic acid, lithocholic acid, ursodeoxycholic acid, taurolithocholic acid, tauroursodeoxycholic acid, taurodeoxycholic acid and glycodeoxycholic acid. For statistical inference, we used a paired *t*-test, and the corresponding *p*-values are shown in each graph. (**D**) Linear relationship between the reduction in plasma LDL-c and the concentration of fecal bile acids (log10 transformed) after alirocumab therapy. Line of best fit for the linear model is plotted with 95% confidence interval (grey shade area). Individual samples are represented with black dots. The regression coefficient, coefficient of determination and the result of the F-test for the linear model are shown on the top of the plot.

Alirocumab treatment was linked to elevated levels of BAs in feces (mean difference (95% CI), 0.20 [0.05–0.34] log10(nmol mg^−1^ feces), paired *t*-test *p* = 0.01) (Figure 2A). This association was driven by a higher proportion of secondary BAs in feces after alirocumab therapy (Figure 2B,C). Interestingly, the levels of fecal BAs in on-treatment samples were correlated with the alirocumab-mediated reduction in plasma LDL-c (β = 0.49, *F*(1,16) = 4.92, *p* = 0.04) (Figure 2D), but not with the decrease in plasma Lp(a) concentration (β = 0.19, *F*(1,16) = 2.73, *p* = 0.12). Conversely, circulating levels of total, primary and secondary BAs, as well as SCFAs were not associated with PCSK9 inhibition (Figures S5–S7). The observed levels in peripheral circulation of total BAs (baseline median (interquartile range (IQR)), 2.33 [1.63–3.42] µM), acetic acid (baseline median (IQR), 121.3 [118.4–122.4] µM), and butyric acid (baseline median (IQR), 0.63 [0.53–0.65] µM), were within the normal range [49,50]. Conversely, propionic acid, which typically exists at an estimated concentration of 4–5 µM in human peripheral blood [50], was found to be substantially elevated in our cohort (baseline median (IQR), 64.20 [63.17–64.36] µM) (Figure S7).

## 4. Discussion

The gut microbiota has emerged as an important contributor to the development of ASCVD, playing a causal role through the production of bioactive metabolites [11,12,13,14]. Previous case–control studies have shown that patients with atherosclerosis have specific gut bacterial profiles characterized by an enrichment of Pseudomonadota (formerly Proteobacteria), including *Enterobacter* spp. and *Escherichia-Shigella*, as well as microorganisms typically associated with the oropharyngeal cavity, such as *Streptococcus* spp. [51,52,53]. Additionally, when compared to healthy controls, patients with ASCVD consistently exhibit a reduction in bacteria that produce SCFAs, like *Akkermansia*, *Roseburia intestinalis*, or *Faecalibacterium prausnitzii*, along with other typical intestinal commensals such as *Bacteroides* spp., *Prevotella copri*, or *Alistipes shahii* [51,52,53]. These ASCVD gut bacterial profiles are associated with a reduced capability to produce SCFAs from the diet, potentially exacerbating the progression of ASCVD [16]. In line with a previous study evaluating the fecal microbiota in patients with coronary heart disease, the fecal bacterial profiles in our cohort were dominated by OTUs belonging to the phylum Bacillota (formerly Firmicutes) [54]. While it is true that four patients in our cohort demonstrated high proportions of OTUs assigned to the *Enterobacter* and the *Escherichia-Shigella* taxa, we did not consistently observe the typical ASCVD-related fecal bacterial profiles across all patients. Notably, our patient cohort exhibited clinical heterogeneity in terms of atherosclerotic phenotypes, including patients with both clinical and subclinical evidence of ASCVD [9]. Additionally, all patients in our study were on long-term statin therapy, which could have potentially confounded the observation of ASCVD-related fecal bacterial profiles. In accordance with a recent study, statins are associated with improvements in fecal bacterial profiles linked to obesity and a reduction in the abundance of bacterial taxa associated with intestinal inflammation [31]. Our findings indicated no taxonomic changes in the fecal microbiota associated with the modulation of cholesterol metabolism using a PCSK9 inhibitor. This observation differs from other cholesterol lowering medications like ezetimibe or statins, which both impact the composition and function of the gut microbiota [30,31,32,33]. Similarly, within our cohort, we did not identify distinct endotypes associated with the fecal bacterial profiles. These results suggests that the benefits associated with the inhibition of PCSK9 are not linked to alterations in the fecal bacterial communities.

Several metabolites produced by gut bacteria such as SCFAs and BAs have been implicated in the pathophysiology of ASCVD [16,55]. Both SCFAs and BAs modulate lipid and cholesterol metabolism, thereby reducing hypercholesterolemia and potentially preventing the onset of atherosclerosis [24,25,26,27,28]. Additionally, cardiomyocytes and endothelial cells express specific receptors for both SCFAs and BAs, allowing these molecules active influence on the cardiovascular function [16,56,57]. Thus, both SCFAs and BAs may prevent ASCVD by modulating blood pressure and maintaining vascular homeostasis [16,58]. Accordingly, a recent phase II study employed a prebiotic intervention, which resulted in a decrease in systolic blood pressure among patients with hypertension [59]. Similarly, another placebo-controlled clinical trial demonstrated that treatment with the secondary BA, ursodeoxycholic acid, improved peak post-ischemic peripheric blood flow in a cohort of patients with chronic heart failure [56]. In our study, PCSK9 inhibition did not show a correlation with the circulating levels of both BAs and SCFAs. These observations are consistent with the unchanged bacterial profiles in feces after treatment with alirocumab. In our cohort, levels of propionic acid were above the estimated levels found in human peripheral blood. Individuals in our study were taking the highest tolerable dose of statins, a medication known to modify metabolic pathways, including the tricarboxylic acid cycle [60], which is essential for the metabolism of propionic acid [61]. Thus, it is plausible that the plasma concentration of propionic acid in our cohort could represent a yet not described pleiotropic effect associated with statin use.

In humans, the excretion of cholesterol predominantly occurs through the fecal route in form of BAs. Interestingly, in our previous study using animal models, we observed that statin therapy was associated with increased levels of BAs in feces [30], suggesting an additional mechanism through which statins exert their cholesterol-lowering effect. In this study, alirocumab treatment was also associated with an elevated concentration of mainly secondary BAs in feces. This observation supports the findings from a previous study utilizing knockout mouse models [62]. The study demonstrated that genetic suppression of PCSK9, in combination with statin therapy, resulted in an increased excretion of BAs in feces [62]. This response was likely a compensatory mechanism to preserve the balance of cholesterol and BAs metabolism in the liver in response to the increased uptake of LDL-c mediated by PCSK9 inhibition [62]. In line with this observation, we found that the concentration of fecal BAs in samples collected during alirocumab therapy was correlated to the alirocumab-mediated reduction in plasma LDL-c. In addition to their primary role in digestion, BAs also have antimicrobial properties [20,21,22], and dysregulation of the metabolism of BAs is linked to the remodelling of the gut microbiota in different human conditions [21]. Interestingly, the increase in the fecal concentration of BAs following alirocumab administration was not associated with a corresponding alteration in the taxonomic composition of the gut microbiota, suggesting that this increment in fecal BAs levels is probably not sufficient to have an impact on the gut bacterial communities.

Our study is significantly limited by the low sample size, which likely affected our ability to detect small effects. Another limitation of our study is the absence of a healthy control cohort, which prevented comparative analysis to determine the presence of typical ASCVD-associated microbial profiles within our patient cohort. Likewise, not including a healthy reference group limited our capacity to differentiate alterations associated with medications such as statins and variations within a typical gut microbial composition. Additionally, we did not include dietary information in our analyses, which constitutes an important factor driving the composition of the gut microbiota. However, patients in our cohort were on long-term treatment with medications such as aspirin, statins, and ezetimibe, which consistently demonstrated a considerable impact on both the composition and function of the gut microbiome [30,31,32,33,63,64,65], comparable to the effects caused by dietary factors or antibiotics [35].

## 5. Conclusions

In summary, we did not observe differences in parameters of microbial ecology before and after treatment with alirocumab. Similarly, circulating levels of the microbial metabolites, BAs and SCFAs, were not affected by therapeutic inhibition of PCSK9. However, we observed an elevated excretion of BAs related to the administration of PCSK9 neutralizing antibodies. This increase in BAs correlated with the extent of LDL-c reduction achieved through alirocumab. The association between PCSK9 inhibition and increased excretion of BAs suggests an additional mechanism contributing to the cholesterol-lowering effects of PCSK9 inhibition.

## Figures and Tables

**Table 1:** Clinical and biochemical characteristics of the 18 patients with elevated Lp(a) concentration at baseline. Data extracted from [9].

	Value
Sex (% males)	61%
Age (years)	56.5 ± 11.2
BMI (kg m^−2^)	28.6 ± 4.4
Systolic blood pressure (mmHg)	124.3 ± 7.3
Diastolic blood pressure (mmHg)	76.3 ± 8.5
Total cholesterol (mmol L^−1^)	3.8 ± 0.6
Triglyceride (mmol L^−1^)	1.1 (0.9, 1.4)
HDL-cholesterol (mmol L^−1^)	1.2 ± 0.3
LDL-cholesterol (mmol L^−1^)	2.1 ± 0.5
ApoB (g L^−1^)	0.8 ± 0.1
Lp(a) (g L^−1^)	1.2 (0.96, 1.44)

Data are presented as mean ± standard deviation (SD), number (%) or geometric mean (95% confidence interval); Apo: apolipoprotein; HDL, high-density lipoprotein; LDL: low-density lipoprotein; Lp(a): lipoprotein(a) BMI, body mass index.

## Data Availability

All the sequencing data generated in this study were deposited in the Sequencing Read Archive under the Bioproject accession PRJNA948889.

## Supplementary Materials

The following supporting information can be downloaded at: https://www.mdpi.com/article/10.3390/microorganisms12010170/s1, Figure S1. Procrustes analyses; Figure S2. Bacterial profiles in fecal samples; Figure S3. Principal component analyses related to Figure 1; Figure S4. Principal component and Procrustes analyses in baseline and on-treatment stool samples; Figure S5. Plasma levels of total bile acids and short chain fatty acids in patients; Figure S6. Plasma levels of primary and secondary bile acids in patients; Figure S7. Levels of specific short chain fatty acids in plasma from patients; Table S1. Taxonomic classification and relative abundance of OTUs classified as Archaea in the unfiltered dataset; Table S2. Absolute read counts for the OTUs identified as potential contaminants by the R package decontam; Table S3. Results of the permutational test of significance of the Procrustes analyses.
