# Supplementary material for: An Analysis of the Gut Microbiota and Related Metabolites following PCSK9 Inhibition in Statin-Treated Patients with Elevated Levels of Lipoprotein(a)
Source: Microorganisms. 2024 Jan 15;12(1):170. doi: 10.3390/microorganisms12010170 (PMC10818477; doi:10.3390/microorganisms12010170)
Supplement: Supplementary file 1 [file microorganisms-12-00170-s001.zip › microorganisms-2762256-supplementary.pdf]

SUPPLEMENTAL MATERIAL

An analysis of the gut microbiota and related metabolites following PCSK9 inhibition in statin-treated patients with elevated levels of lipoprotein(a)

Jose A. Caparrós-Martín, Patrice Maher, Natalie C. Ward, Montserrat Saladié, Patricia Agudelo-Romero, Stephen M. Stick, Dick C. Chan, Gerald F. Watts and Fergal O'Gara

Table S1. Taxonomic classification and relative abundance of OTUs classified as Archaea in the unfiltered dataset.

OTU	Relative abundance
Archaea;Euryarchaeota;Methanobacteria;Methanobacteriales;Methanobacteriaceae;Methanobacterium;	0.000154
Archaea;Euryarchaeota;Methanobacteria;Methanobacteriales;Methanobacteriaceae;Methanobrevibacter;	0.00596
Archaea;Euryarchaeota;Methanobacteria;Methanobacteriales;Methanobacteriaceae;Methanosphaera;	0.000606
Archaea;Euryarchaeota;Methanomicrobia;Methanosarcinales;Methanoperedenaceae;Candidatus Methanoperedens;	0
Archaea;Euryarchaeota;Methanomicrobia;Methanosarcinales;Methanosaetaceae;Methanosaeta;	0.0000852
Archaea;Euryarchaeota;Thermoplasmata;Methanomassiliicoccales;Methanomethylophilaceae;uncultured;	0.00153

Table S2. Absolute read counts for the OTUs identified as potential contaminants by the R package decontam. BAL specimens are identified by a numeric code. The last row represents a negative extraction control.

	Bacteria; Firmicutes; Bacilli; Bacillales; Staphylococcaceae; Staphylococcus;	Bacteria; Proteobacteria; Alphaproteobacteria; Rhizobiales; Beijerinckiaceae; Bosea;	Bacteria; Proteobacteria; Alphaproteobacteria; Rhizobiales; Rhizobiaceae; Ochrobactrum;
**S9.WK5**	0	0	0
**S9.WK18**	0	0	0
**S7.WK5**	0	0	0
**S7.WK18**	0	0	0
**S4.WK5**	0	0	0
**S4.WK18**	0	0	0
**S32.WK5**	0	0	0
**S32.WK18**	0	0	0
**S3.WK5**	0	0	0
**S3.WK18**	0	0	0
**S29.WK5**	0	0	0
**S29WK18**	0	0	0
**S25.WK5**	0	2	0
**S25.WK18**	0	0	0
**S24.WK5**	0	0	9
**S24.WK18**	0	0	0
**S23.WK5**	2	0	0
**S23.WK18**	0	0	0

**S2.WK5**	0	0	0
**S2.WK18**	0	0	0
**S18.WK5**	0	0	0
**S18.WK18**	0	0	0
**S17.WK5**	0	2	0
**S17.WK18**	2	0	0
**S16.WK5**	0	2	3
**S16.WK18**	0	0	0
**S15.WK5**	0	0	0
**S15.WK18**	0	0	0
**S12.WK5**	0	0	0
**S12.WK18**	0	0	0
**S11.WK5**	6	0	0
**S11.WK18**	8	0	0
**S10.WK5**	0	0	0
**S10.WK18**	0	0	0
**S1.WK5**	0	0	0
**S1.WK18**	0	0	0
**NEG**	407	413	489

Table S3. Results of the permutational test of significance of the Procrustes analyses. The high correlation coefficient and low value of the goodness-of-fit statistic M^2, indicates a great concordance between the compared datasets and suggests that the filtering steps did not significantly impact the structure of the original dataset (Raw counts).

**Datasets**	**Procrustes Sum of Squares (M^2)**	**Correlation in a symmetric Procrustes rotation**	**Significance**
Raw counts Vs Removed low counts/singletons/Unclassified/Mithocondria/ Chloroplast/Eukaryota/Acinetobacter	0.000000005	1	0.001
Raw counts Vs Removed low counts/singletons/Unclassified/Mithocondria/ Chloroplast/Eukaryota/Acinetobacter and contaminants	0.000000005	1	0.001

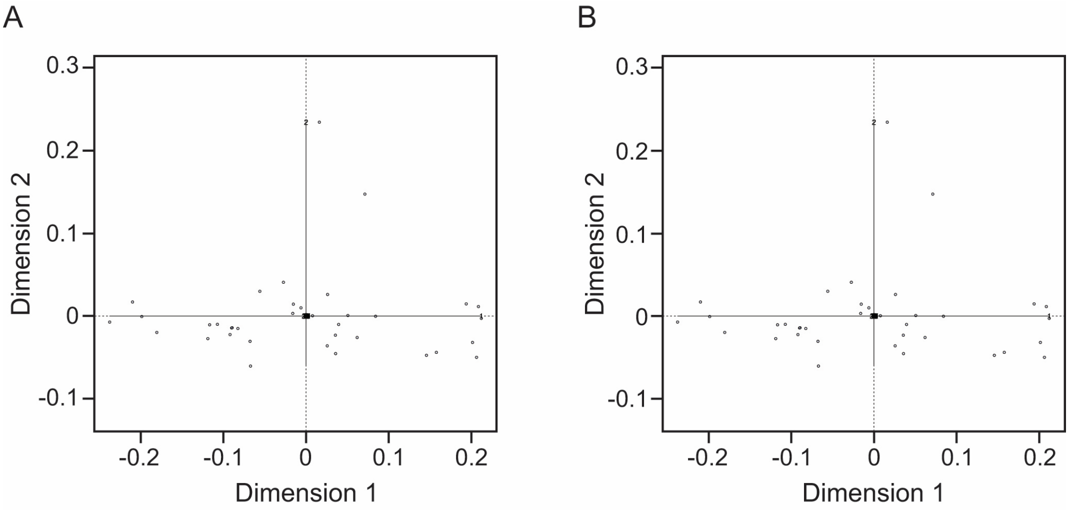

Figure S1. Procrustes analyses. A. Procrustes analysis showing the correlation between the bacterial profiles in the unfiltered dataset (raw data) and the bacterial profiles after removing low count taxa, singletons, unclassified reads and reads classified as Mitochondria, Chloroplast, Eukarya or *Acinetobacter*. **B.** Procrustes analysis comparing the taxonomic profiles between the bacterial profiles in the unfiltered dataset (raw data), and the dataset after removing low count taxa, singletons, unclassified reads, reads classified as Mitochondria, Chloroplast, Eukarya or *Acinetobacter* as well as the three OTUs identified as putative contaminants shown in Table S1. Solid black lines illustrate the required rotation of the indicated axes to match the samples (circles) from the first ordination (filtered dataset) into the second ordination (unfiltered dataset).

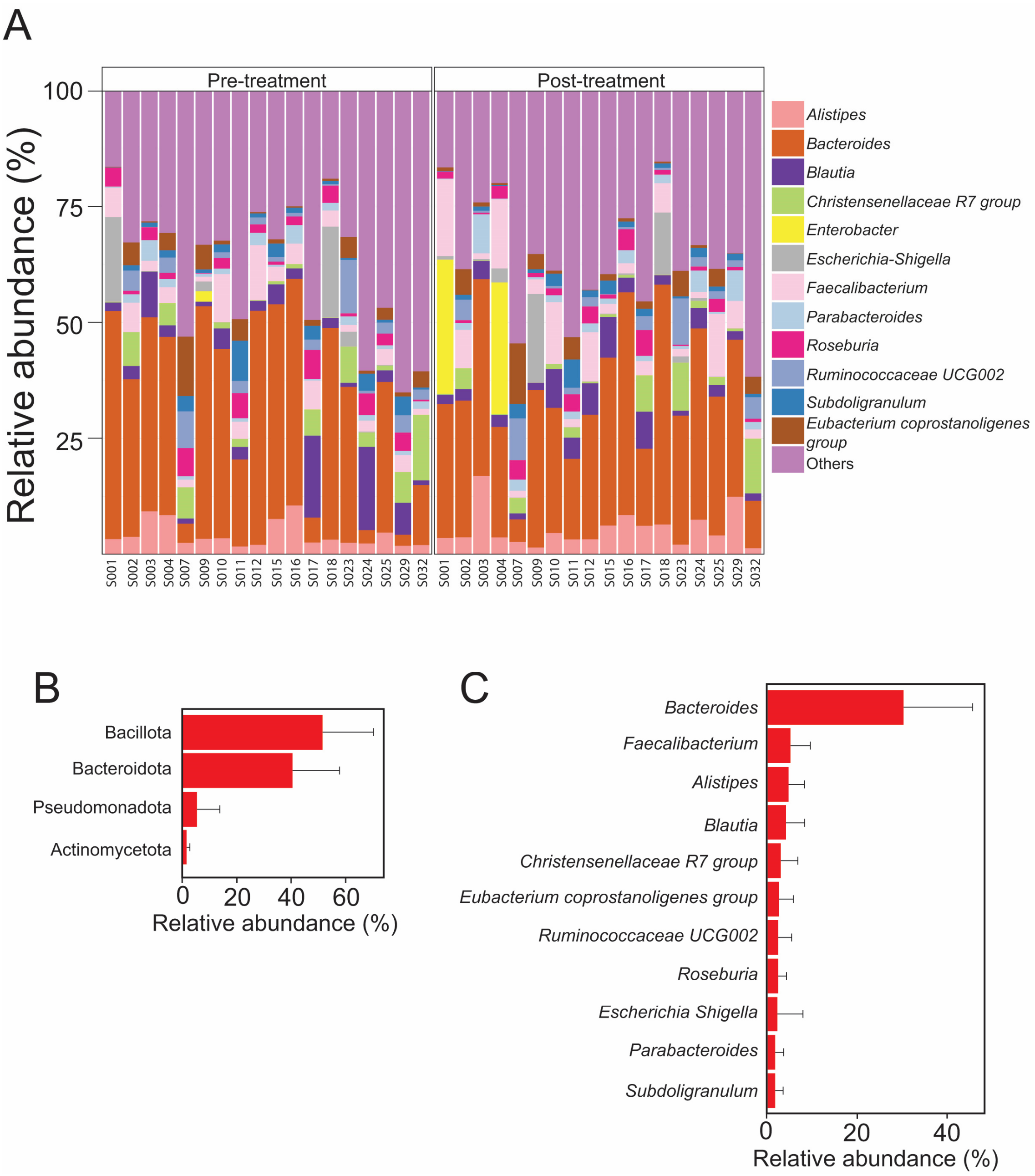

Figure S2. Bacterial profiles in fecal samples. A. Bacterial compositional profiles in the cohort of fecal samples evaluated in this study. The 11 more abundant OTUs are represented. Fecal profiles are grouped by collection point. **B-C.** Barplots representing the mean relative abundance and the standard deviation of the top more abundant taxonomic entities in our cohort of fecal specimens at phylum (**B**) and genus level (**C**).

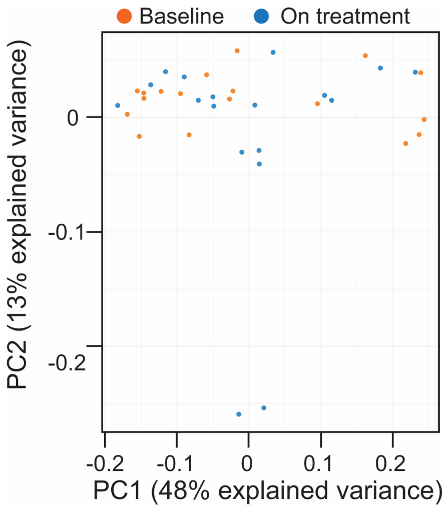

Figure S3. Principal component analysis related to figure 1. Principal component analysis displays the projection of bacterial stool community profiles (centered log-ratio transformed) in a two-dimensional space. Each dot represents an independent sample, color-coded to differentiate between baseline (orange) and on-treatment (blue) timepoints. Principal component 2 (PC2) separates two on-treatment samples away from the rest of the samples, being an OTU assigned to the *Enterobacter* taxon, the main driver of variation along this component. The two specific samples clustering away had a high proportion of *Enterobacter* detected (see Figure S1A, yellow box).

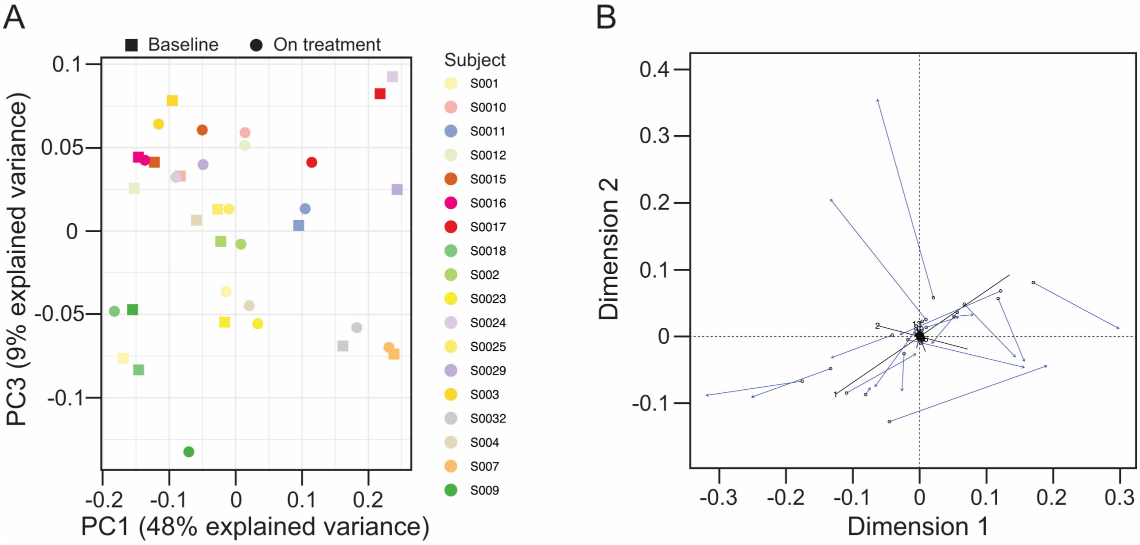

Figure S4. Principal component and Procrustes analyses in baseline and on-treatment stool samples. A. Principal component analysis showing the projection of the compositional profiles of the bacterial stool communities (centered log-ratio transformed) in a two-dimensional space. Individual samples collected at baseline (square) or on-treatment (circle) are coloured based on the subject ID. **B.** Procrustes analyses comparing the taxonomic profiles between baseline and on-treatment. Solid black lines illustrate the required rotation of the indicated axes to match the samples (circles) from the first ordination (baseline) into the second ordination (on-treatment), with blue arrows pointing to the spatial location of the samples in the second ordination (on-treatment).

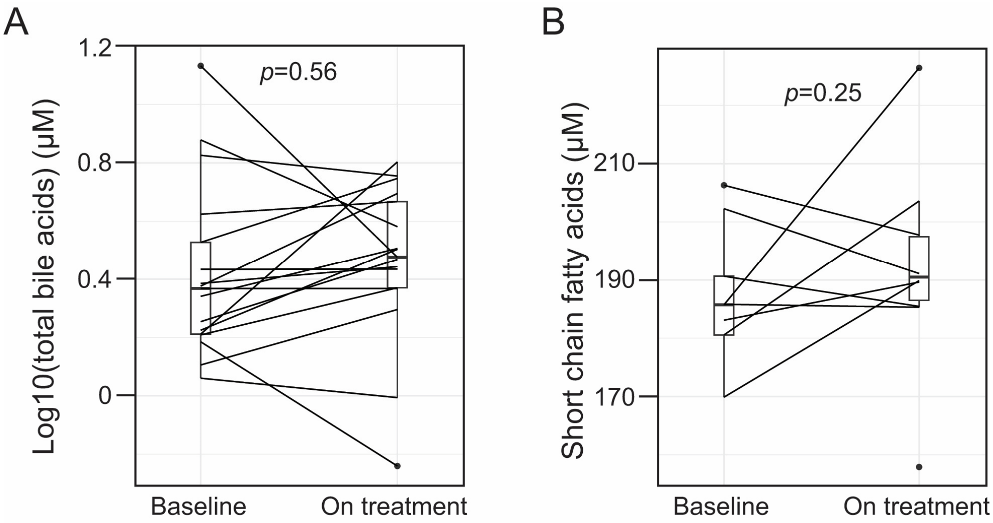

Figure S5. Plasma levels of total bile acids and short chain fatty acids in patients. A-B. Boxplots representing the concentration of bile acids (log10 transformed) (**A**), and short chain fatty acids (**B**) in paired plasma samples collected at baseline and on-treatment. Samples from the same patient are connected using straight lines. Paired t-test was used for statistical inference and the corresponding *p*-values are shown in each graph.

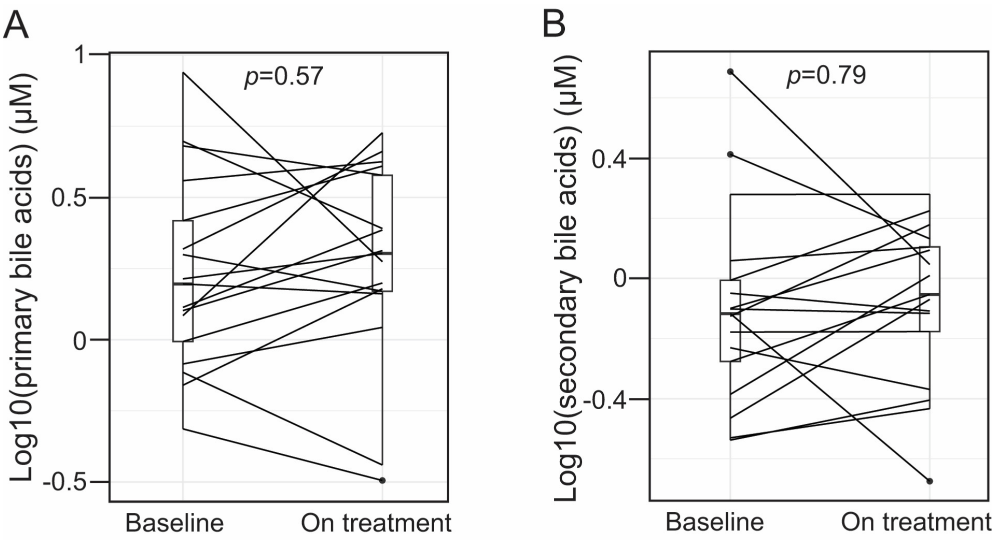

Figure S6. Plasma levels of primary and secondary bile acids in patients. A-B. Boxplots representing the concentration of primary (**A**) and secondary (**B**) bile acids (log10 transformed) in paired plasma samples collected at baseline and on-treatment. Samples from the same patient are connected using straight lines. Paired t-test was used for statistical inference and the corresponding *p*-values are shown in each graph.

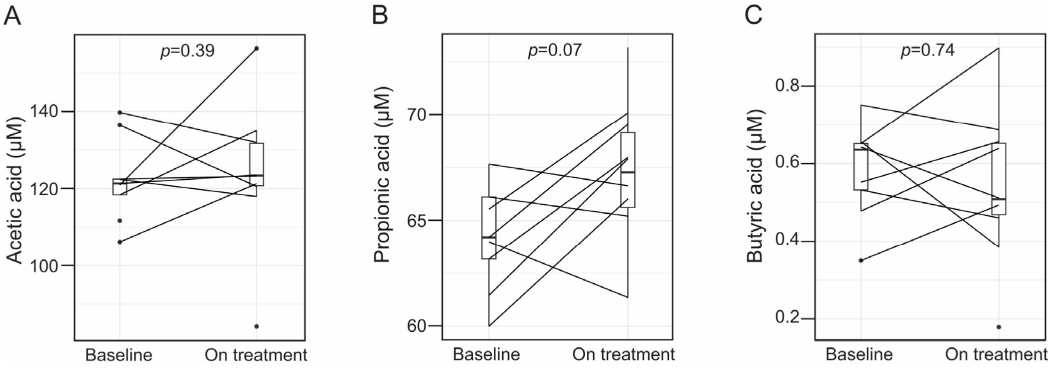

Figure S7. Levels of specific short chain fatty acids in plasma from patients. A-B. Boxplots representing the concentration of acetic acid (**A**), propionic acid (**B**) and butyric acid (**C**) in paired plasma samples collected at baseline and on-treatment. Samples from the same patient are connected using straight lines. Paired t-test was used for statistical inference and the corresponding *p*-values are shown in each graph.
